# High-contrast X-ray micro-radiography and micro-CT of ex-vivo soft tissue murine organs utilizing ethanol fixation and large area photon-counting detector

**DOI:** 10.1038/srep30385

**Published:** 2016-07-27

**Authors:** Jan Dudak, Jan Zemlicka, Jakub Karch, Matej Patzelt, Jana Mrzilkova, Petr Zach, Zuzana Hermanova, Jiri Kvacek, Frantisek Krejci

**Affiliations:** 1 Institute of Experimental and Applied Physics, Czech Technical University in Prague, Horska 3a/22, 128 00 Prague, Czech Republic; 2 Faculty of Biomedical Engineering, Czech Technical University in Prague, Namesti Sitna 3105, 272 01 Kladno, Czech Republic; 3 Third Faculty of Medicine, Charles University in Prague, Ruska 87, 100 00 Prague, Czech Republic; 4 National Museum, Vaclavske namesti 68, 115 79 Prague, Czech Republic

## Abstract

Using dedicated contrast agents high-quality X-ray imaging of soft tissue structures with isotropic micrometre resolution has become feasible. This technique is frequently titled as virtual histology as it allows production of slices of tissue without destroying the sample. The use of contrast agents is, however, often an irreversible time-consuming procedure and despite the non-destructive principle of X-ray imaging, the sample is usually no longer usable for other research methods. In this work we present the application of recently developed large-area photon counting detector for high resolution X-ray micro-radiography and micro-tomography of whole *ex-vivo* ethanol-preserved mouse organs. The photon counting detectors provide dark-current-free quantum-counting operation enabling acquisition of data with virtually unlimited contrast-to-noise ratio (CNR). Thanks to the very high CNR even ethanol-only preserved soft-tissue samples without addition of any contrast agent can be visualized in great detail. As ethanol preservation is one of the standard steps of tissue fixation for histology, the presented method can open a way for widespread use of micro-CT with all its advantages for routine 3D non-destructive soft-tissue visualisation.

Study of micro- and macroscopic structure of biological tissue provides essential information required both in medical diagnostics and research. Existing techniques for observation of tissue morphology, morphometry and phenotyping at the microscopic scale rely on histology^1^. Conventional histology, however, requires time consuming, complicated and destructive sample preparation protocols. The sample processing typically consists of different types of tissue dehydration, fixation, staining and resin-embedding followed by precise slicing and mounting on glass slides. Individual sample slices are then observed using a microscope, images are digitalized, evaluated and stored. Spatial resolution within the slice is basically given by the wavelength of used light. Improving further the spatial resolution requires the use of electron microscopy^1^. As each tissue sample is processed into a set of slices, the histology provides a sort of 3D information about the studied sample. Nevertheless, the spatial resolution of the obtained data set is highly anisotropic since the thickness of slices (affecting the resolution in the Z axis) is much larger than the spatial resolution within a single slice (XY plane). The assembling of the volumetric information from the set of 2D slices is, moreover, a complicated time consuming procedure prone to different artefacts^2^.

## Volumetric methods in conventional histology

A histological method producing true volumetric information was introduced as High Resolution Episcopic 3D Microscopy (HREM) or Surface Imaging Microscopy (SIM). HREM and SIM have much in common with conventional histology, but utilizing a different scanning approach isotropic spatial resolution down to 0.5 μm can be achieved^2^,^3^,^4^,^5^. A significant drawback of these methods is, however, that they destroy the sample completely. On the contrary, in the case of conventional histology glass slides with sample slices can be stored and used repeatedly (for microscopy or more importantly for other techniques). In general, the optical-based methods can provide ultimate spatial resolution and contrast down to the cellular level, they can render 3D information about the investigated sample, but these techniques are destructive to the sample.

## Virtual histology by means of X-ray imaging techniques

With the rapid development of technology in the field of X-ray imaging, high resolution information about the inner structures of objects can be obtained even non-destructively. The spatial resolution of X-ray micro-tomography systems is becoming almost comparable to conventional histology (tens of nanometres in the case of synchrotron radiation sources and micrometre level in the case of laboratory-scale devices^6^,^7^). Computed tomography, furthermore, produces a virtual 3D voxel-based model of the sample with isotropic spatial resolution. The reconstructed volume can be rendered and sliced in any required plane giving much more freedom in sample observation and data evaluation than conventional histology. Thanks to the non-destructive approach of the method, samples remain intact and still fully usable for further investigation procedures.

In spite of the high spatial resolution provided by X-ray micro-radiography and micro-CT, visualisation of soft tissue remains a challenging task due to the very low intrinsic attenuation contrast between the investigated structures. Consequently, for imaging of soft tissue with X-rays the application of a high-Z contrast agent is usually necessary^8^,^9^,^10^,^11^,^12^,^13^,^14^,^15^. To obtain a specific affinity of dedicated contrast agents to various tissue structures, a proper sample preparation protocol must be then followed. These contrast staining procedures are, however, elaborate, often time consuming (up to several days) and irreversible which prevents the use of a sample for other research methods.

## Photon counting detector technology

The steady progress in miniaturization of front-end electronics in the last decades has opened the possibility for development of semiconductor pixel X-ray detectors with sophisticated digital per-pixel signal processing functionality enabling detection by so-called photon counting. Several different photon counting imaging detectors (PCD) have been developed in the last decade which are today commercially available (Medipix and Timepix^16^, PILATUS^17^ and Eiger^18^, XPAD^19^ and PiXirad^20^). These detectors operate typically in dark-current-free mode in which the signal generated by each incoming X-ray photon is processed at the pixel level individually including pre-amplification, comparison with the pre-adjusted amplitude threshold (set above the pixel intrinsic noise) and digital counting. As a result of this signal processing, these detectors are practically noiseless and the obtained contrast-to-noise ratio in X-ray radiographs and CT scans is limited only by the number of detected X-ray photons. Consequently, when applied for X-ray absorption radiography, even structures showing low difference in attenuation (e.g. soft tissue) can be visualised with high CNR compared to conventional imaging detectors utilizing scintillating sensors and charge-coupled detection instead of photon counting^21^.

Despite the significant progress in imaging parameters provided by the photon counting technology a crucial limitation in the practical use of these devices for imaging has been the small sensitive area of only few square centimetres. This technological barrier has been fully overcome by the development of large-area photon counting semiconductor detectors such as the WidePIX device^22^ consisting of a matrix array of Timepix chips equipped with edgeless sensors tightly placed side by side using customized tiling technique. The assembled detector matrix array has practically no gaps between chips resulting in a fully sensitive area of up to 14.3 × 14.3 cm^2^ (array of 100 Timepix chips). Such large-area semiconductor pixel detectors combined with modern table-top sources (e.g. micro-focus X-ray tubes) open the way for extended and new applications in X-ray imaging.

In this work we demonstrate performance of this recently developed large area hybrid pixel photon counting detector for high resolution X-ray microradiography and micro-tomography of *ex-vivo* ethanol-only preserved murine organs. The effect of *ex-vivo* soft tissue contrast enhancement after ethanol-preservation has been demonstrated in studies related to phase contrast X-ray imaging^23^,^24^. However, in the case of techniques based on X-ray absorption, ethanol is generally used as a soft tissue fixative and the sample needs to be further stained by a contrast agent (Lugol solution, phosphotungstic acid, osmium tetroxide, etc.)^8^,^9^,^10^,^11^,^12^,^13^,^14^. The presented experimental measurements demonstrate that using the PCD it is possible to visualize fine structures in ethanol-preserved mouse tissue samples even without use of any further contrast staining. The approach, furthermore, preserves the sample for standard imaging techniques, such as histology. The presented approach, therefore, radically simplifies the soft-tissue sample processing protocol for X-ray micro-radiography and micro-CT and opens the way for widespread use of these techniques for routine 3D non-destructive *ex-vivo* soft tissue visualisation.

## Results

**Figure 1:**
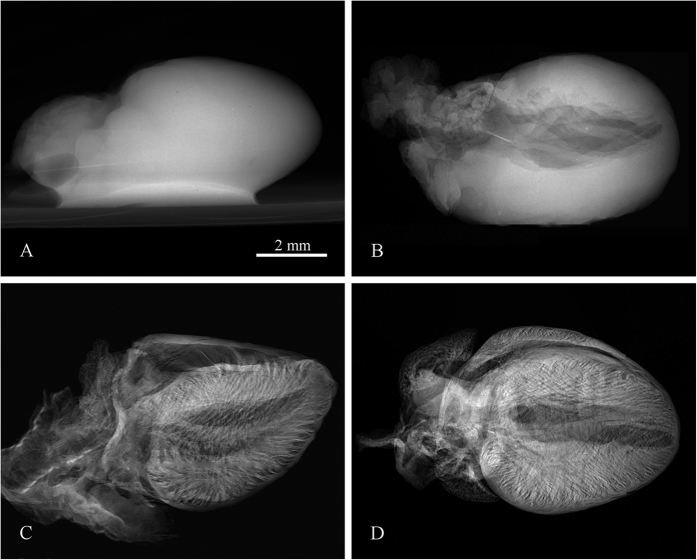
Demonstration of the contrast improvement in X-ray micro-radiography between a native sample and ethanol preserved samples. X-ray radiography of a native mouse heart (**A**) and samples preserved in 50% ethanol solution (**B**), 97% ethanol solution (**C**) and the ethanol series of increasing concentrations (**D**). Acquisition parameters: Tube voltage 60 kV, current 120 μA, acquisition time 40 s.

Compared to water, ethanol evaporates more effectively promptly leaving all kinds of cavities such as ventricles, vessels, alveoli, bronchi etc. Structures normally filled by liquid, therefore, become hollow and produce detectable absorption contrast. Since ethanol stiffens the tissue, investigated organ samples can easily withstand time consuming tomographic scans (from tens of minutes up to hours) without significant changes of the tissue shape and inner structure. The demonstration of the observed contrast improvement of the ethanol preserved tissue sample compared to the native sample is shown in Fig. 1. Microradiography of the native heart kept in saline (Fig. 1A) provides minimal contrast of inner structures as ventricles and veins are still filled by blood or saline having almost the same attenuation properties as the surrounding heart muscle tissue. On the other hand, in the case of ethanol preserved hearts (Fig. 1B–D) the microradiography reveals numerous details of sample inner structures. In all tested samples the gained contrast developed during first days of fixation, as ethanol penetrated the tissue, however, after 7 days in ethanol solution the contrast becomes stable without further changes. All samples presented in this work were preserved for at least 7 days.

From three different ways of ethanol fixation which were tested (50% solution, 97% solution, and series of increasing concentration 50–97%), we evaluated as most appropriate the approach based on the series of increasing ethanol concentration. The 50% ethanol solution has maintained the native look of the tissue, but it did not stiffen and improved the contrast of samples sufficiently. On the other hand, the use of the high ethanol concentration (97%) directly to a native tissue sample has resulted in severe tissue deformation or even occurrence of ruptures.

**Figure 2:**
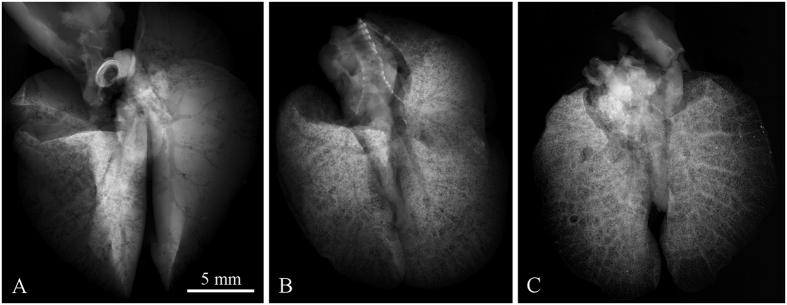
Demonstration of contrast development in X-ray micro-radiography of ethanol-preserved mouse lungs as a function of resting time. Three different samples scanned after 10, 20 and 40 minutes of relaxation, respectively, are shown. Short resting time makes the trachea and bronchial tree being visible (**A**), while longer resting time leads to discernment of the alveolar structures of lungs. In (**B**) a superposition of bronchial and alveolar structures are visible, in (**C**) the alveolar structures hinder the visibility of the trachea and bronchial tree. Acquisition parameters: Tube voltage 60 kV, current 120 μA, acquisition time 40 s.

Experimental measurements indicate that the contrast develops with respect to the degree of drying of the sample. The ethanol evaporation is the fastest from the surface of the sample and from larger cavities. Contrast in radiographic images changes consequently as a function of resting time (the time delay between sample removal from the ethanol solution and the measurement) within the same type of tissue, as demonstrated for the case of lungs in Fig. 2. Different lung samples A, B and C were extracted from the ethanol solution and rested on paper towel in air for varying time periods. While sample A rested for just 10 minutes, it shows mostly the trachea and its bifurcations. At longer rest time, finer structures start appearing (see Fig. 2B). Finally the contrast of trachea and bronchial tree is almost completely shadowed by pronounced alveolar structure (see Fig. 2C).

**Figure 3:**
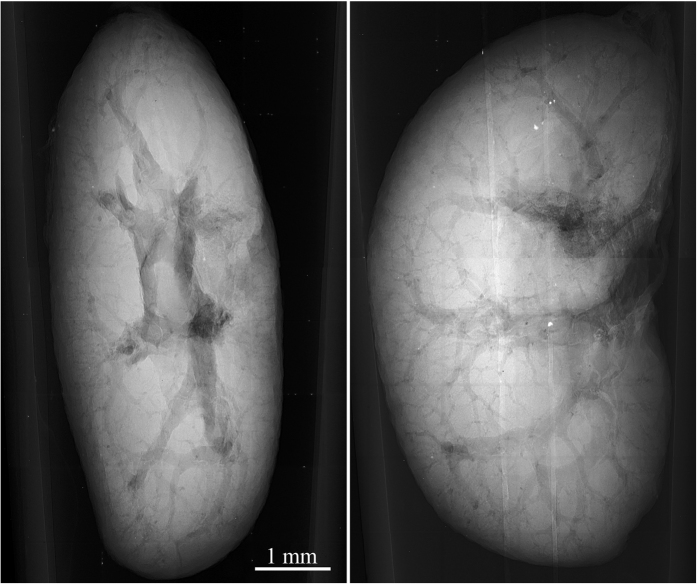
Selected projections from a tomographic dataset of ethanol-preserved murine kidney acquired using the high resolution micro-tomography setup equipped with the large-area PCD. The images demonstrate possibility to visualise various inner structures with superb image quality just based on the ethanol preservation. Acquisition parameters: Accelerating voltage 60 kV, current 90 μA, acquisition time 10 s.

**Figure 4:**
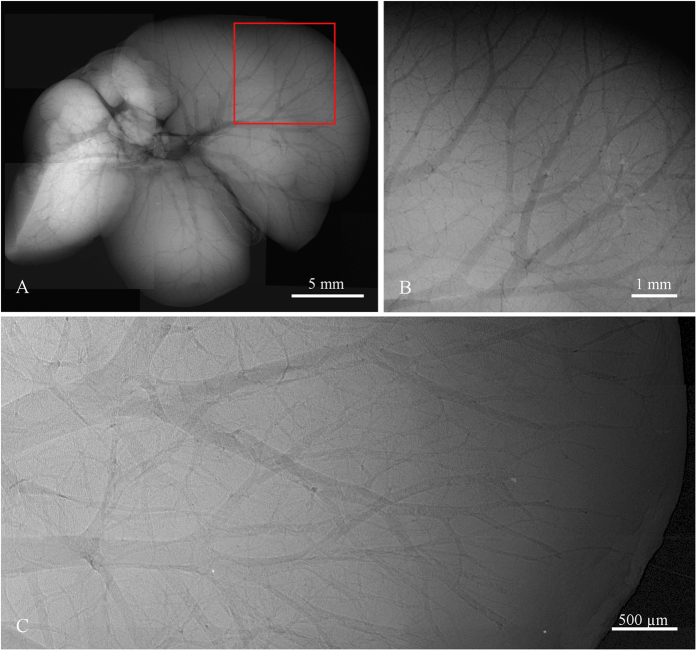
High resolution X-ray radiography of ethanol preserved mouse liver scanned using the compact micro-CT scanner with Quad detector assembly (**A,B**) and the high resolution setup (**C**). Due to the limited FOV the presented micro-radiographic image (**A**) is merged from 9 individual tiles. The selected ROI (**B**) shows the fine vascular structure of the liver lobe with venules diameter down to 40 μm. ROI of liver with 5 μm spatial resolution acquired using the large area Timepix detector (**C**) enables revealing venular structures down to 15 μm. Note also a white brim along the outer border of capillaries formed by phase effects enhancing further the final contrast. Acquisition parameters: Tube voltage 60 kV, current 120 μA, acquisition time 40 s.

**Figure 5:**
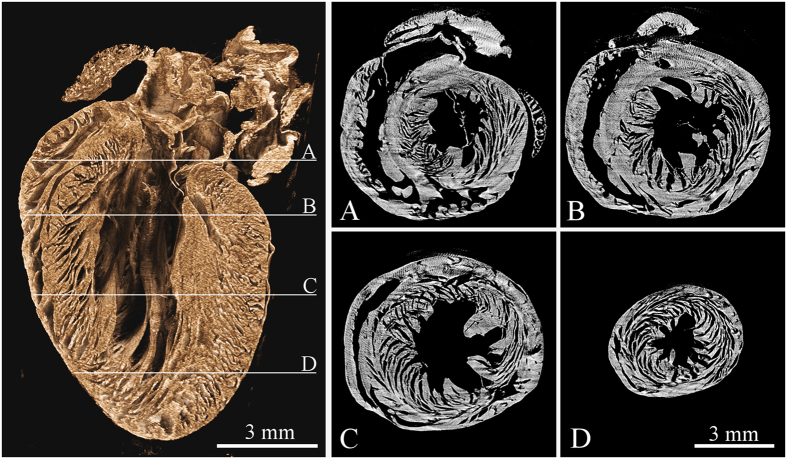
Tomographic reconstruction of a mouse heart acquired using the high resolution setup and large area Timepix detector. The left image shows the volume rendering of reconstructed dataset visualized using the false-colour system. The right part of the figure shows four different transversal slices (see labels A thru D) across the reconstructed volume demonstrating the heart vortex – helical structure of the muscle fibres. See supplementary files 1 and 2 providing animations of the reconstructed data in two different planes. Acquisition parameters: Tube voltage 70 kV, current 100 μA, 720 projections, acquisition time 5 s. per projection. Spatial resolution 7.2 μm.

In a similar manner also other hollow systems like veins, arteries and various ventricles provide extensive contrast enhancement following alcohol staining. Contrast enhancement in a murine kidney is demonstrated in Fig. 3. High contrast was obtained in the case of liver, where the vessel system is clearly pronounced down to the 15 μm thick venules, see Fig. 4. The dehydration also improves the detectability of tissue structures such as muscle fibres as the muscle tissue reacts differently than the surrounding fascial layer of muscle (visible in Fig. 1C,D). In the case of micro-tomography of the heart this effect can be used for visualization of the heart vortex – heart wall with helically shaped muscle fibres (see Fig. 5 and supplementary files 1 and 2). The data for the presented tomographic reconstruction was acquired using the high resolution setup equipped with the large area PCD. Thanks to the magnifying geometry used for the scan, the spatial resolution of projections is 7.2 μm. The transversal slices of the reconstructed 3-D model (see right part of Fig. 5) clearly reveal the helical structure of the heart vortex. Beside that it is possible to observe inner structures like heart chambers or valves, and to perform various distance, surface and volume measurements and other common analyses.

## Discussion

The proposed approach provides enhanced CNR imaging with 3D information about investigated soft tissue structures at the micrometre scale without physical sectioning of the sample. Quick, non-destructive and non-distorted 3D visualisation of soft tissue structures with spatial resolution approaching the cellular level opens new possibilities in so-called virtual histology^8^,^9^. The spatial resolution, in our case limited to 5 μm by the focal spot size of the X-ray source used, can be further improved by new table-top laboratory sources with focal spots size well below 1 μm.

Very good soft tissue contrast together with non-destructive volumetric information can be provided also by highly sophisticated methods such as X-ray phase-contrast^23^,^24^,^25^,^26^,^27^,^28^, dark-field imaging^29^,^30^ or X-ray microscopy^31^,^32^. These methods, however, require an advanced setup and very special X-ray beam characteristics, which in most of the cases can be found only at large scale synchrotron facilities. These requirements crucially limit further widespread application of these techniques, e.g. in biology.

Currently used X-ray micro-CT staining techniques for *ex-vivo* soft tissue absorption imaging utilize high-Z contrast agents which are delivered to the specific structure by elaborate, time demanding and irreversible staining techniques which depend also on the knowledge of skilled staff. Using the proposed method, high quality micro-radiographs and micro-CT scans of basically all murine organs can be acquired just after simple ethanol preservation of tissue. The method, moreover, keeps the samples fully usable for further measurements and research. The significant simplification of the sample preparation protocol and compatibility with established techniques makes consequently the method easily accessible to the general scientific community and opens the use of micro-CT as a new standard tool for inspection of soft tissue biological samples.

**Figure 6:**
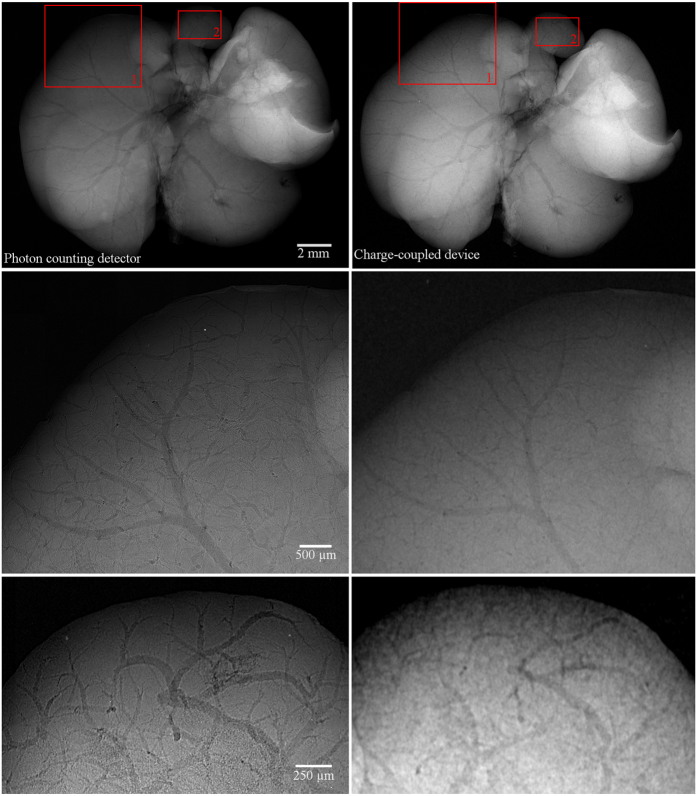
Comparison of X-ray micro-radiography of the ethanol-preserved mouse liver acquired using the PCD (left column) and CCD (right column). The pixel resolution of both setups was set to 4.3 μm. While globally both images look very similar, when observing a small region of interest (lower rows) the PCD reveals finer structures thanks to much higher CNR. Acquisition parameters were 60 kV, 100 in the case of the high resolution set-up with PCD and 40 kV, 260 μA in the case of the CCD-based setup. Acquisition time of each imaging system was set individually to provide comparable detected intensity of the open beam. While the PCD clearly visualizes venules smaller than 15 μm, in the case of the CCD detector only structures larger than ca. 60 μm are visible.

A set of comparative measurements was performed using a commercially available micro-CT scanner Bruker 1172 equipped with 11 Mpixel CCD detector (9 um pixel pitch) and micro-focus X-ray tube. The comparison has showed that the contrast improvement of soft tissue structures based on the ethanol staining is detectable even with state-of-the-art conventional X-ray cameras. Nevertheless, the profit of a noiseless PCD lies in the possibility to detect even extremely small variation in density/thickness formed, for example, by few micron thin veins. For charge-integrating devices such sensitivity is a difficult task due to various intrinsic sources of noise (dark current, leakage current, read-out noise) negatively affecting the CNR of radiographic images. Results of comparison between the large area PCD and CCD camera are demonstrated for the case of ethanol-preserved murine liver in Fig. 6. The pixel resolution of micro-radiographic systems was set to 4.3 μm in both cases and comparable detected open beam intensity was used. The comparison shows that PCD clearly provides better results in terms of CNR which provides significantly improved visualisation of many faintly attenuation object features. Moreover, CNR of the PCD radiograph can be further improved just by exposure time prolongation. The detail visibility in radiographs acquired using PCD is also partially positively affected by their steep point spread function.

Another important advantage of the approach based on the large-area photon counting detector is the capability to image whole murine organs with very high magnification. The sensitive area of state-of-the-art Charge-Coupled-Devices (CCDs) which are a promising detector technology currently used in micro-CT scanners is still limited to few square centimetres and their use is therefore limited to imaging of small objects. Thanks to development of large area photon counting detectors even larger organs, such as human, can be also visualized with moderate spatial resolution given basically by the pixel size of the detector (55 μm). The approach therefore provides from a single tomographic scan very complex 3-D model of relatively large tissue volume. This feature together with the high contrast provided by the ethanol staining opens new possibilities in understanding of the structure and functionality of soft tissue organs.

## Conclusions

We have developed and demonstrated a flexible imaging method for 3D imaging of *ex-vivo* soft tissue samples based on the use of the large area photon-counting detector and simple ethanol preservation. The method provides substantial improvement of image quality of various soft tissue structures in absorption X-ray micro-radiography and micro-tomography applied without the use of higher-Z contrast agents. The achieved contrast provides different type of information compared to conventionally used contrast agents. Moreover, ethanol preservation stabilizes the samples structurally and improves the stability of sample imaging during long lasting tomographic scans. As ethanol preservation is one of standard steps of tissue fixation for histology, the presented approach can open a way for widespread use of micro-CT with all its advantages for routine 3D non-destructive soft tissue visualisation.

## Methods

### Sample processing

**Figure 7:**
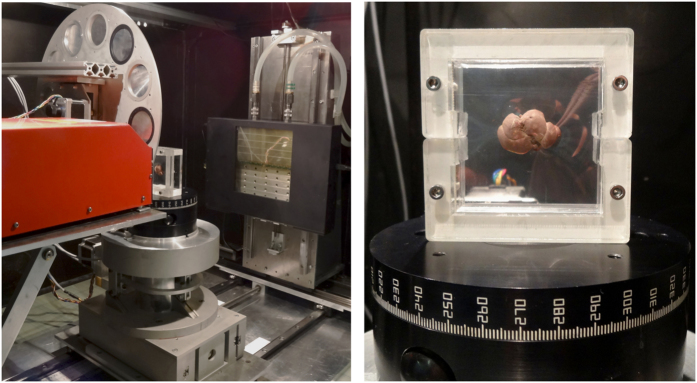
High-resolution table-top X-ray imaging setup. The system is equipped with a micro-focus X-ray tube, rotating carrousel with calibrating foils, precise sample positioning stage and the large area photon counting detector with sensitive area 14.3 × 7.15 cm^2^ (left). Detail view of a sample holder used for 2D high-resolution X-ray imaging of soft biological samples (right).

A set of measurements with *ex-vivo* mouse organs was performed. Use of laboratory animals was approved by Ethical Committee of the Third Faculty of Medicine, Charles University in Prague. The animals were treated with accordance to guidelines defined by Ethical Committee in decisions no. 246/1992 and no. 419/2012. Genetically modified mice C57BL/6 (weight 17 g) were terminated by ether inhalation overdose and their organs (brain, heart, kidney, liver and lungs) were extracted out for purposes of this study. All samples were inserted into ethanol solution immediately after extraction. As fixative we used 50% and 97% ethanol solutions and an ethanol series with gradually increasing concentration (50–97%). The samples were scanned after 24 hours, 72 hours, 1 week and 2 weeks after inserting into the fixative. Before data acquisition each sample was rested on the air for certain time (5–60 minutes) to let the redundant ethanol evaporate from the surface of the sample and from cavities. For the purposes of 2D projection imaging the sample was placed in a sample holder keeping the sample in place between two thin self-adhesive stretch foils which minimised the occurrence of motion artefacts and slowed down the further drying-out of the tissue (see Fig. 7 right). In the case of tomographic acquisition, samples were fit into the properly-sized cylindrical double-compartment sample holder with an ethanol reservoir that fixed the sample mechanically and kept saturated gaseous atmosphere preventing structural changes of the samples^33^.

### X-ray micro-CT s3etup and measurements

For the first evaluation, samples were scanned using a compact small animal X-ray micro-CT scanner^34^. The scanner provides spatial resolution ca. 28 μm and limited field of view given by the detector size (photon counting Timepix Quad detector^16^ with 300 μm thick Si sensor, 512 × 512 pixels, 55 μm pixel pitch, sensitive area 7.92 cm^2^). Thanks to the rotating gantry construction with a short source-to-detector distance and high beam intensity, rapid measurements of unstable samples and acquiring of tomographic datasets consisting of many projections are feasible. The gantry housing the X-ray source and detector rotates around the sample laying stationary on the holder in the horizontal direction. The configuration minimises the possibility of changes within the soft biological sample during the scan.

In order to obtain high-resolution radiographs and micro-CT scans of whole murine organs the upgraded high-resolution X-ray imaging setup^35^ at IEAP CTU in Prague has been used. The high-resolution setup is equipped with the Hamamatsu L8601-01 X-ray tube with 5 μm focal spot and the large area photon counting detector WidePIX_10×5_ composed of 50 Timepix chips^22^ providing the total pixel resolution of 2560 by 1280 pixels (see Fig. 7 left). The basic detector assembly (a single detector tile) consists of an edgeless 300 μm thick silicon sensor bump-bonded to a CMOS pixelated Timepix read-out chip (256 by 256 pixels, 55 μm pixel pitch)^16^. The high-resolution system is placed inside of a shielded vault enabling the use of imaging geometry with high magnification factor. The precise motorised positioning system allows scanning large samples in multiple sub-acquisitions that virtually increase the detector sensitive area. The best achievable spatial resolution is slightly below 5 μm (limited by the penumbral effect of the tube spot size).

The detected intensity in acquired data was over 10^5^ and 1.5·10^4^ events per pixel behind the object for 2D radiography and tomographic projection, respectively. All presented 2D radiographies and the projections used for tomographic reconstruction were processed by beam hardening correction^36^. The X-ray spectrum used for measurements corresponded to a tungsten anode with thin beryllium output window operated at 50–70 kVp. No filtration was used to maintain maximal content of soft X-rays providing the highest contrast in faintly attenuating soft tissue organs. The 2D radiographies were acquired with the highest possible magnification and resolution: ca. 28 μm and 5 μm in the case of the compact micro-CT scanner and the high resolution setup, respectively. The magnification used for tomographic measurements was set with respect to the dimensions of the sample to optimally cover the detector area. The tomographic data was reconstructed using the Volex reconstruction engine based on the filtered back-projection (Fraunhofer-Allianz Vision, Germany), alternatively an in-house implemented OSEM based iterative reconstruction algorithm was used also. Ring filtering was performed as a part of data pre-processing in sinogram domain using combined wavelet-Fourier filtering^37^. The reconstructed volumetric data was visualized using open source volume renderers AMIDE^38^ and CTVox^39^.

## Additional Information

**How to cite this article**: Dudak, J. *et al.* High-contrast X-ray micro-radiography and micro-CT of ex-vivo soft tissue murine organs utilizing ethanol fixation and large area photon-counting detector. *Sci. Rep.* **6**, 30385; doi: 10.1038/srep30385 (2016).

## Supplementary Material

Supplementary Video 1

Supplementary Video 2

Supplementary Information
