# Supplementary material for: High-contrast X-ray micro-radiography and micro-CT of ex-vivo soft tissue murine organs utilizing ethanol fixation and large area photon-counting detector
Source: Sci Rep. 2016 Jul 27;6:30385. doi: 10.1038/srep30385 (PMC4961961; doi:10.1038/srep30385)
Supplement: Supplementary Information [file srep30385-s3.pdf]

High-contrast X-ray micro-radiography and micro-CT of ex-vivo soft tissue murine organs utilizing ethanol fixation and large area photon-counting detector – Supplementary Information

Jan Dudak[1,2], Jan Zemlicka[1], Jakub Karch[1,2], Matej Patzelt[3], Jana Mrzilkova[3], Petr Zach[3], Zuzana Hermanova[4], Jiri Kvacek[4] and Frantisek Krejci[1*]

[1.] Institute of Experimental and Applied Physics, Czech Technical University in Prague
Horska 3a/22, 128 00 Prague, Czech Republic

[2.] Faculty of Biomedical Engineering, Czech Technical University in Prague
Namesti Sitna 3105, 272 01 Kladno, Czech Republic

[3.] Third Faculty of Medicine, Charles University in Prague
Ruska 87, 100 00 Prague, Czech Republic

[4.] National Museum, Vaclavske namesti 68, 115 79 Prague, Czech Republic

[*] Corresponding author [e-mail: frantisek.krejci@utef.cvut.cz]

Supplementary video 1: Tomographic reconstruction of a mouse heart acquired using the high resolution setup equipped with the large area photon counting detector and presented ethanol-preservation technique. The video shows different axial slices across the reconstructed volume demonstrating the heart vortex – helical structure of the muscle fibres in heart wall. Acquisition parameters: Tube voltage 70 kV, current 100 μA, 720 projections, acquisition time 5 s per projection. Spatial resolution 7.2 μm.

Supplementary video 2: Tomographic reconstruction of a mouse heart acquired using the high resolution setup equipped with the large area photon counting detector and presented ethanol-preservation technique. The video shows different sagittal slices across the reconstructed volume. The data clearly demonstrate the possibility to observe inner structures like heart ventricles, valves or tendinous chords and to perform various distance, surface and volume measurements at the micrometre level. Acquisition parameters are the same as for Supplementary video 1.
